# Fractionated SRT using VMAT and Gamma Knife for brain metastases and gliomas — a planning study

**DOI:** 10.1120/jacmp.v16i6.5255

**Published:** 2015-11-08

**Authors:** Marie Huss, Pierre Barsoum, Ernest Dodoo, Georges Sinclair, Iuliana Toma‐Dasu

**Affiliations:** ^1^ Medical Radiation Physics Stockholm University Stockholm Sweden; ^2^ Department of Medical Physics Karolinska University Hospital Stockholm Sweden; ^3^ Department of Nerosurgery Karolinska University Hospital Stockholm Sweden; ^4^ Medical Radiation Physics Stockholm University and Karolinska Institute Stockholm Sweden

**Keywords:** radiosurgery, fractionation, Gamma Knife, VMAT

## Abstract

Stereotactic radiosurgery using Gamma Knife (GK) or linear accelerators has been used for decades to treat brain tumors in one fraction. A new positioning system, Extend™, was introduced by Elekta AB for fractionated stereotactic radiotherapy (SRT) with GK. Another option for fractionated SRT is advanced planning and delivery using linacs and volumetric modulated arc therapy (VMAT). This project aims to assess the performance of GK Extend™ for delivering fractionated SRT by comparing GK treatments plans for brain targets performed using Leksell GammaPlan (LGP) with VMAT treatment plans. Several targets were considered for the planning: simulated metastasis‐ and glioma‐like targets surrounding an organ at risk (OAR), as well as three clinical cases of metastases. Physical parameters such as conformity, gradient index, dose to OARs, and brain volume receiving doses above the threshold associated with risk of damaging healthy tissue, were determined and compared for the treatment plans. The results showed that GK produced better dose distributions for target volumes below 15 cm^3^, while VMAT results in better dose conformity to the target and lower doses to the OARs in case of fractionated treatments for large or irregular volumes. The volume receiving doses above a threshold associated with increased risk of damage to normal brain tissue was also smaller for VMAT. The GK consistently performed better than VMAT in producing a lower dose‐bath to the brain. The above is subjected only to margin‐dependent fractionated radiotherapy (CTV/PTV). The results of this study could lead to clinically significant decisions regarding the choice of the radiotherapy technique for brain targets.

PACS numbers: 87.53.Ly, 87.55.D‐

## I. INTRODUCTION

Stereotactic radiosurgery (SRS) using Gamma Knife (GK) (Elekta AB, Stockholm, Sweden) in which the dose of radiation is delivered in a single fraction is one of the main treatment modalities used in the management of intracranial primary tumors and brain metastases.^(1)^ The current use of intracranial SRS is, however, limited to small lesions due to the associated increased risk for complications to the tissue in the healthy brain which increases with irradiated volume and dose.^(2)^ For larger targets, fractionated stereotactic radiation therapy (FSRT) using linear accelerators is usually performed based on encouraging studies showing high efficacy and low toxicity of FSRT compared to SRS.^3^, ^4^ Thus, at Karolinska University Hospital, Stockholm, Sweden, the routine practice is to treat with single fraction GK SRS targets up to a maximum diameter of 3 cm, or a maximum volume of $10-12\;{\text{cm}}^{3}$. Larger targets are assessed for microsurgery as a first hand approach. In case the patient is not able to undergo microsurgery, FSRT using linear accelerators (margin dependent on CTV/PTV) or Gamma Knife by stereotactic frame fixation (nonmargin dependent, no CTV/PTV) becomes the second best alternative.

The recent introduction by Elekta of the Extend™ immobilization system compatible with Leksell Gamma Knife^®^ Perfexion™ has opened another FSRT‐alternative for the treatment of larger targets by GK. Currently, the Extend™ immobilization system is not in current use at the Karolinska University Hospital. Therefore, this project aimed to study the performance of GK using Extend™ and linac‐based volumetric‐modulated arc therapy (VMAT) for delivering FSRT to brain metastases and recurrent gliomas, two common brain lesions treated with SRS. The project also aimed to compare the Leksell GammaPlan (LGP) plans with treatment plans performed using Eclipse planning system (Varian Medical Systems, Palo Alto, CA) with respect to target coverage and dose to organs‐at‐risk (OARs) and normal tissue.

## II. MATERIALS AND METHODS

### A. Patient characteristics

**Figure 1:**
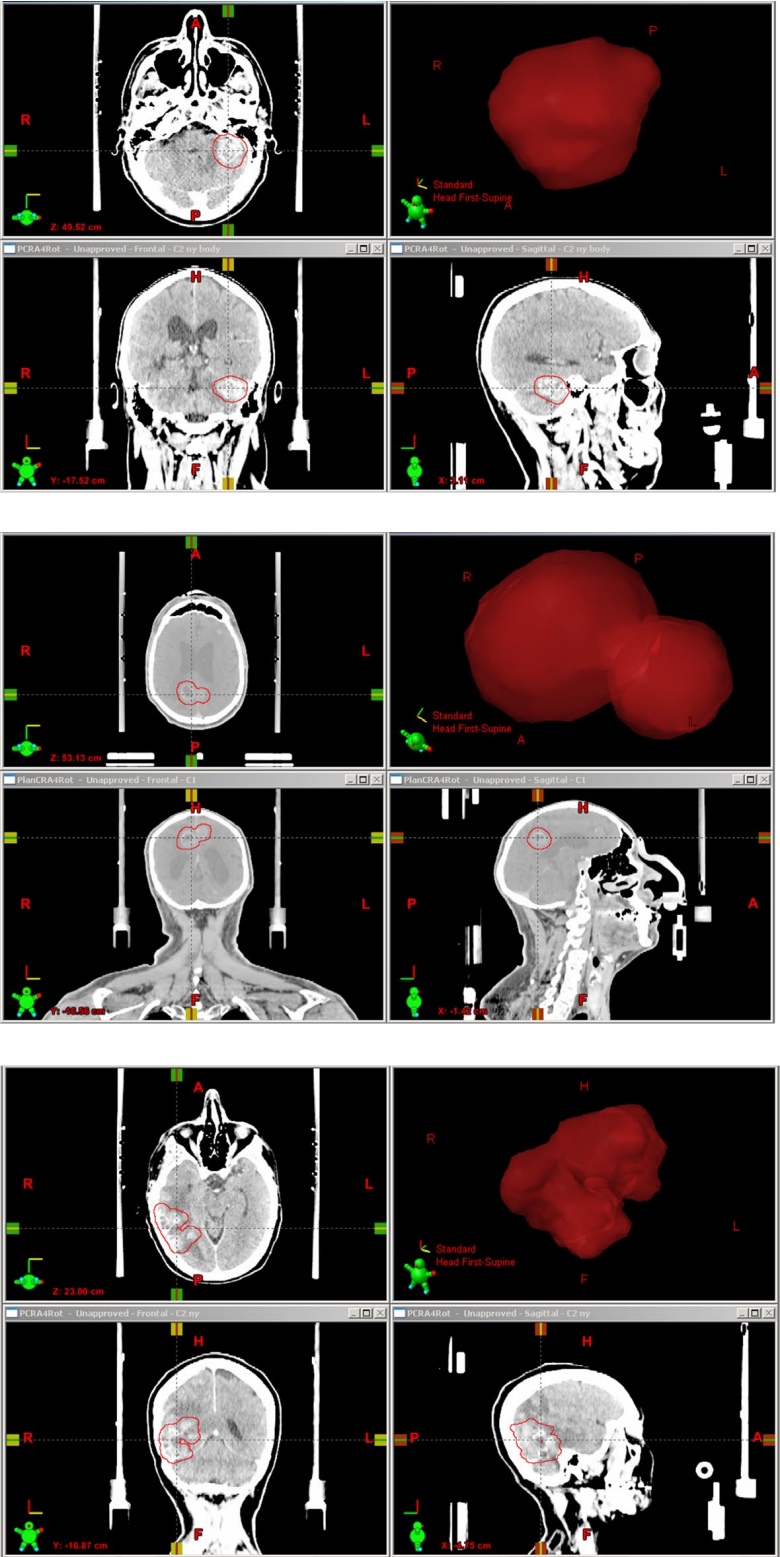
Central transversal, coronal, and sagittal sections showing the extent and the location of the brain metastases, as well as the 3D model of the target, for the patients included in this study. The upper panel corresponds to Patient 1 in Table 1, the middle and the lower panels corresponding to Patient 2 and Patient 3, respectively.

Three patients previously treated with VMAT at the Karolinska University Hospital for brain metastases were included in the study. Patient characteristics indicating the tumor volume are given in Table 1. The location and the extent of the metastases for the three patients are shown in Fig. 1. The fixation system used for the three patients receiving FSRT for brain metastases, HeadFIX^®^ (Elekta AB, Stockholm, Sweden), is very similar to the Extend™ system.

**Table 1:** Patient characteristics for the three patients with brain metastases treated with VMAT, the fractionated schedules and threshold doses indicating the biologically equivalent 10 Gy‐volume

*Patient No.*	*Volume (cm^3^)*	*Fractionation Scheme*	*Threshold Doses Indicating the Biologically Equivalent 10 Gy‐volume*
1	19.5	10×4.2 Gy	26 Gy
2	23.8	5×7 Gy	20 Gy
3	58.8	7×6 Gy	23 Gy

### B. Simulated targets for FSRT

In order to investigate the influence of the size and shape of the target on the quality of the GK and VMAT plans, two classes of brain targets were simulated.

The first class, metastasis‐like targets, was simulated as rounded structures in the shape of ellipsoids. The dimensions, in terms of lateral, longitudinal, and transversal diameter are indicated in Table 2. The volumes of the metastasis‐like simulated targets range over a broad interval of values from 1.1 to 44.6 cm^3^.

**Figure 2:**
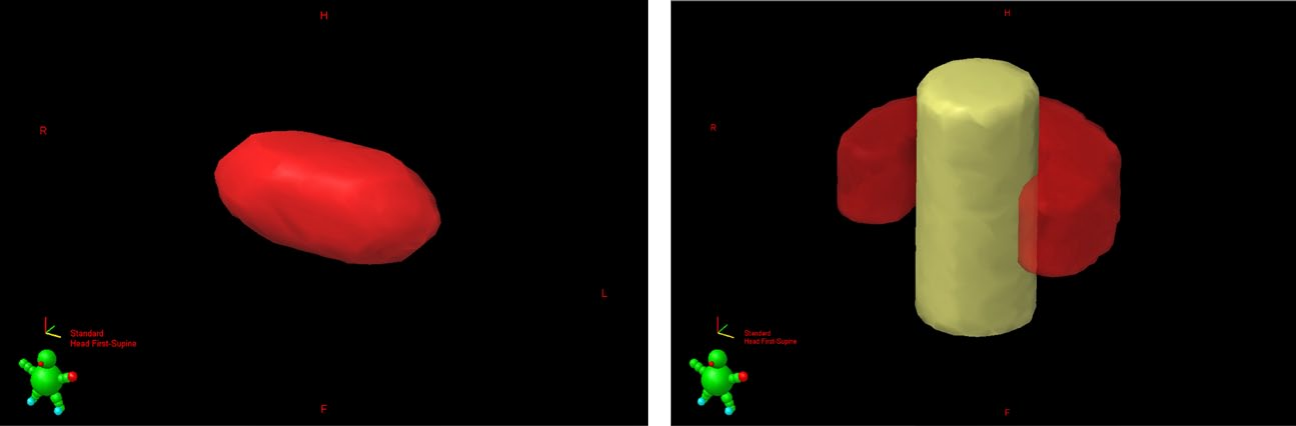
Examples of the 3D representations of the simulated metastases (left) and glioma‐like target (red) surrounding the OAR (yellow) (right).

The second class, glioma‐like targets surrounding an OAR, was simulated as half‐ring–shaped targets surrounding a cylindrical OAR. The characteristics of the glioma‐like targets are also indicated in Table 2. Figure 2 shows a 3D representation of one metastasis and one of the gliomas, together with the corresponding OAR.

**Table 2:** Characteristics of the simulated metastases and gliomas

*Metastases Target*	*Lateral Length (cm)*	*Longitudinal Length (cm)*	*Transversal Length (cm)*	*Volume (cm^3^)*
Metastases 1	2.0	1.0	1.0	1.1
Metastases 2	3.0	1.4	1.4	3.5
Metastases 3	4.0	2.0	2.0	8.5
Metastases 4	5.0	2.4	2.4	15.3
Metastases 5	6.0	3.0	3.0	29.6
Metastases 6	7.0	3.4	3.4	44.6
*Glioma Target*	*Maximum Length (cm)*			*Volume (cm^3^)*
Glioma 1	4.2			5.4
Glioma 2	5.1			13.0
Glioma 3	5.9			23.5

### C. Estimation of necessary PTV margins

One of the key features defining SRS delivered in one fraction is the absence of margins when delineating the target. However, in case of FSRT using GK or VMAT, one has to take into account the possible setup errors and allow for margins around the clinical target volume (CTV), and thus determine the planning target volume (PTV). In the present study, the PTV margins were determined applying the method, proposed by van Herk et al.,^(5)^ which guarantees that 90% of patients in the population receive a minimum cumulative CTV dose of at least 95% of the prescribed dose. The estimated PTV margin based on positioning errors for the three patients included in this study was 2.5 mm, and the same PTV was used for both the GK and the VMAT plans.

### D. Dose prescription

The simulated metastasis‐like targets were prescribed 7×6 Gy and the glioma‐like targets 8×5 Gy, respectively. The prescribed fractionated doses to each clinical case of patients with brain metastases are shown in Table 1, together with the patient characteristics.

### E. Treatment planning for GK

The GK plans were performed in the Leksell Gamma Plan (LGP) version 10.0 (Elekta AB). The planning details for the metastasis‐like and glioma‐like simulated targets, as well as for the three clinical cases, are given in Table 3. The isodose levels for which the dose should be prescribed are based on a prospective study showing that, for larger target volumes, dose plans should preferably be made at isodose levels between 35%–60% in order to simultaneously achieve high target coverage, high selectivity, gradient index (GI) <3 and reasonable treatment times. The selectivity was defined and evaluated as the ratio of the overlap between the PIV and TV to the PIV, where *PIV* is the prescription isodose volume and *TV* is the target volume:

(1) $$S=\frac{PIV\cap TV}{PIV}$$

A selectivity score of unity, or 100%, ensures that no tissue outside the target will be irradiated with the prescribed dose.

**Table 3:** The GK planning details for the metastases, glioma‐like simulated targets, and the three clinical cases

*Target*	*Normalization Isodose Level (%)*	*Selectivity (%)*	*Time/Fraction (min)*	*Equivalent 10 Gy‐volume In Brain (cm^3^)*
Metastasis 1	40	87	13	3
Metastasis 2	35	89	18	9
Metastasis 3	35	88	13	22
Metastasis 4	40	87	20	39
Metastasis 5	40	93	24	71
Metastasis 6	60	91	28	105
Glioma 1	50	62	39	20
Glioma 2	40	63	26	53
Glioma 3	50	82	25	72
Patient 1	35	88	17	36
Patient 2	35	89	30	60
Patient 3	40	82	42	123

### F. VMAT treatment planning

VMAT planning was performed in the Eclipse treatment planning system version 8.8 (Varian Medical Systems). The details of the parameters and the optimization approach used in this study are given in Table 4.

**Table 4:** Description of the optimization approach and summary of the parameters used for VMAT planning

	*Dist. to Border (cm)*	*Start Dose (%)*	*End Dose (%)*	*Falloff*	*Priority*	*Adj. during MR Level* ^b^
*Normal Tissue Objective*	*0*	*100*	*20*	*0.15*	*150*	
Dose limiting annulus UO^a^	50% of prescribed dose to 50% of dose limiting annulus (DLA)				125	2, 3
PTV LO^a^	98% of prescribed dose to 100% of target				50	4, 5
OAR UO	0% of maximum tolerable dose to 100% of OAR				50 or 125	2, 3
PTV first UO	150% of prescribed dose to 0% of target				50	4, 5
PTV second UO	98% of prescribed dose to 100% of target				0	
MUs	Maximum 2000				50	
No of arc	4 arcs:					
	360° couch rotation 0°					
	180° couch rotation 45°					
	180° couch rotation 90°					
	180° couch rotation 135°					
Collimator rotation	Alternate 45° or 135°					
Jaw setting	Maximum diameter of target plus 10 mm					
Beam energy	6 MV					

a Upper/Lower Objective — the volume that should receive a maximum/minimum dose in the target.

b Multiresolution level in the optimization process.

### G. Evaluation of the plans and parameters used for comparison between the performances of GK and VMAT

The GK and VMAT plans were compared based on dose‐volume histograms (DVHs) for the PTV, normal tissue, and OARs.

Further comparisons were performed with respect to the following dosimetric and volumetric parameters: mean and maximal doses to the PTV, volumes receiving 1–10 Gy, 10–20 Gy, 20–30 Gy, and 30–40 Gy, respectively, the low dose‐bath to the skull evaluated as the volume receiving 1–5 Gy, and doses to OARs.

The gradient index (GI)^(6)^ and Paddick conformity index (PCI)^7^, ^8^ were also determined and compared for the GK and VMAT plans.

The Paddick conformity index (PCI) was calculated as:

(2) $$PCI=\frac{{\left(PIV\cap TV\right)}^{2}}{TV\cdot PIV}$$

A PCI of unity will correspond to a perfectly conformal plan. A low PCI indicates poor conformity, but it does not allow for distinguishing between under‐ and overtreatment. A PCI of 50%, for example, could correspond to a plan with only 50% coverage of the target, or a plan with only 50% of the PIV inside the target.

To compare treatment plans of equal PCI, the dose gradient index (GI) could be used:

(3) $$GI=\frac{PIV_{ISO/2}}{PIV}$$

where $PIV_{ISO/2}$ is the prescription isodose volume enclosing half the prescription dose.

An additional parameter, named equivalent 10 Gy‐volume, was also used for comparison based on common practice at Karolinska University Hospital. Thus, the 10 Gy‐volume is a parameter that is taken into account when planning GK treatments, since it is associated with risk of damages to normal tissue. For fractionated treatments, however, one has to determine the equivalent biological doses that will indicate the threshold doses for defining the volumes of the brain associated with the risk of damage.

Thus, the equivalent biological doses in case of fractionated treatments, given in a number of fractions different than one, were calculated using the concept of biological effective dose (BED) proposed by Barendsen.^(9)^ The volume receiving the critical dose associated with the risk of damage or more, will be called the equivalent 10 Gy‐volume throughout this report.

BED for a given fractionation schedule in which the dose is delivered in n fractions of size d, is calculated as:

(4) $$BED=n\cdot d\left(1+\frac{d}{\alpha /\beta }\right)$$

where *α/β* ratio is dependent on tissue being equal to 2 Gy for brain tissue and brainstem as an OAR.^(10)^

The total dose given in a defined number of fractions indicating the equivalent 10 Gy‐volume in 1 fraction can be calculated by assuming that the BED values for the single dose and the fractionated schedule are the same.

(5) $$D=n\left(-\frac{\alpha /\beta }{2}+\sqrt{{\left(\frac{\alpha /\beta }{2}\right)}^{2}+\frac{\alpha /\beta \cdot BED_{s\text{ingle}}}{n}}\right)$$

The threshold doses indicating the biologically equivalent 10 Gy‐volume for the fractionated schedules used for the three clinical cases of metastases are shown in Table 1, together with the patient characteristics. The corresponding threshold dose calculated with Eq. (5) for the fractionated schedule used for gliomas (8×5 Gy fractions) is 24 Gy. The fractionation schedule of 7 fractions of 6 Gy used for the simulated metastases results in a corresponding threshold dose of 23 Gy. Using this approach, the equivalent 10 Gy‐volume was assessed for each of the plans and fractionation schedule, and the GK and VMAT plans were compared with respect to it.

## III. RESULTS

**Figure 3:**
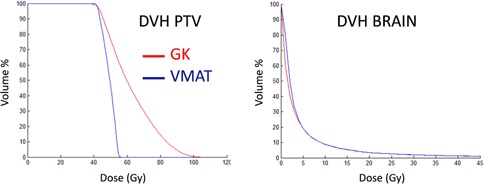
DVHs for the PTV (left) and brain (right) for a representative metastases‐like target (no. 4 in Table 2).

**Figure 4:**
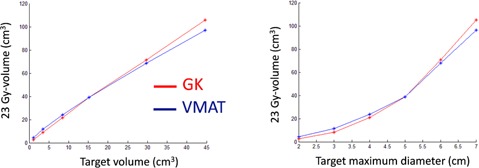
The equivalent 10 Gy‐volume (23 Gy‐volume) as a function of target volume (left) and maximum target diameter (right) for the six metastases.

**Figure 5:**
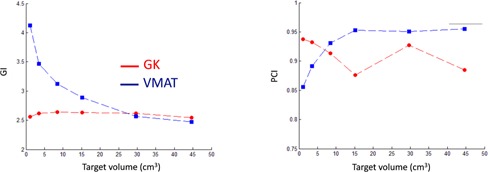
GI (left) and PCI (right) for the six metastases.

**Figure 6:**
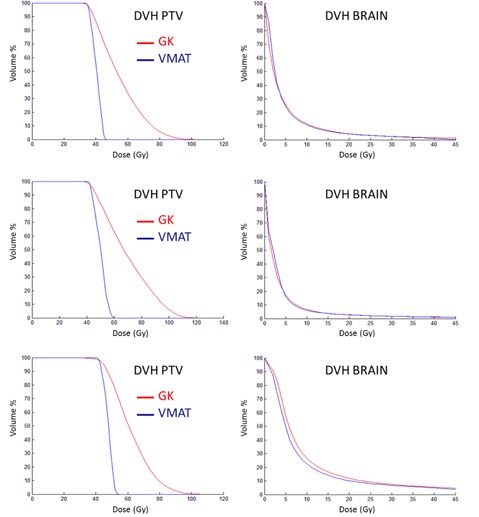
DVHs for the PTV (left panel) and brain (right panel) for the patient cases considered in this study (Patient 1, top panels; Patient 2, middle panels; Patient 3, bottom panels).

The comparison between the treatment plans for GK and VMAT for the metastasis‐like simulated targets shows a general trend of larger maximum dose and inhomogeneity in the target for the GK plan. The dose distributions in the brain, presented as DVHs, are very similar for the two modalities. One representative example for all six simulated metastases, corresponding to metastasis no. 4 in Table 2, is shown in Fig. 3. The equivalent 10 Gy‐volume as a function of target volume and the maximum target diameter for the six metastases are shown in Fig. 4. GI and PCI for the six metastases obtained with the GK and VMAT are shown in Fig. 5. Very similar results were found for the three clinical cases of patients with brain metastases. Figure 6 shows the larger maximum dose and inhomogeneity in the target for the GK plan and the almost coinciding dose distributions in the brain for all patients considered in this study.

**Figure 7:**
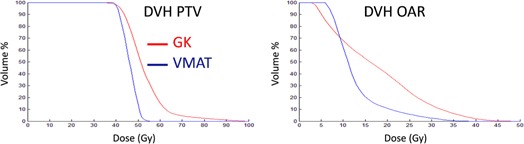
DVHs for the PTV (left) and brain (right) for a representative glioma‐like target (no. 2 in Table 3).

**Figure 8:**
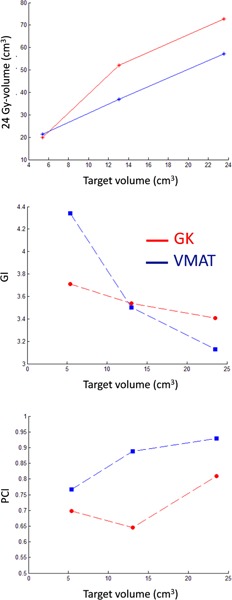
The equivalent 10 Gy‐volume (24 Gy‐volume) as a function of target volume (upper panel), GI (middle panel), and PCI (lower panel) for the glioma‐like targets obtained with the GK and VMAT.

The same trend of larger maximum dose and inhomogeneity in the target for the GK plan in comparison to VMAT observed for the previously discussed targets was also seen for gliomas. Example DVHs for PTV and OAR in the two compared treatment plans of a glioma‐like target (no. 2 in Table 2), irregular in shape surrounding an OAR, are shown in Fig. 7. Figure 8 shows the results of the comparison between plans expressed as the equivalent 10 Gy‐volume in brain, GI, and PCI for all the glioma‐like targets. One could observe that plan conformity is higher for VMAT, which is also better at sparing the OAR compared to the GK. The OAR volume that receives doses up to about 10 Gy are smaller for the GK but, for higher doses, the VMAT plan is able to spare the OAR to a larger extent.

**Figure 9:**
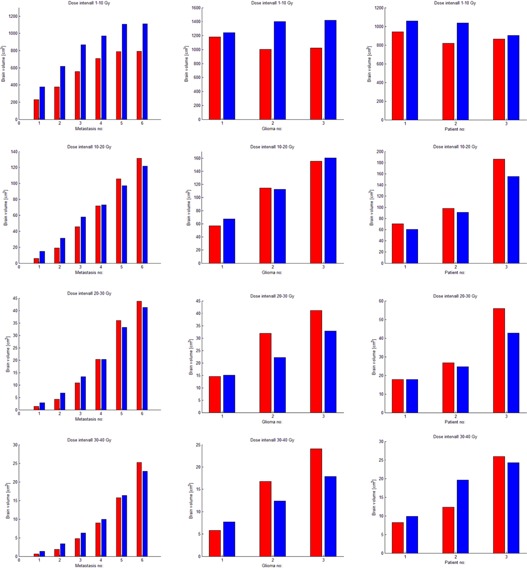
Brain volume receiving doses between 1‐10 Gy, 10‐20 Gy, 20‐30 Gy, and 30–40 Gy for the GK (red bars) and VMAT(blue bars) for the six metastases (left column), the three glioma‐like targets (middle column), and clinical cases (right column).

An extensive comparison between the brain volumes receiving doses between 1–10 Gy, 10–20 Gy, 20–30 Gy, and 30–40 Gy for the GK and VMAT plans for all for the six metastases, the three glioma‐like targets, and clinical cases, is shown in Fig. 9. For all the metastases, regardless their size, the volume of the brain receiving doses between 1–10 Gy is consistently larger for the VMAT plans, while the volumes receiving 10–20 Gy, 20–30 Gy, and 30–40 Gy are larger for the GK plans. However, it has to be mentioned that those volumes receiving higher doses are much smaller in absolute values than the ones receiving lower doses. Similar trends have been observed for almost all the other cases included in this analysis.

## IV. DISCUSSION

The potential of VMAT to deliver SRS plans for intracranial targets has recently been explored by several research groups and results have already been reported in the literature.^11^, ^12^, ^13^, ^14^, ^15^, ^16^ However, only very few of these studies involved a direct comparison between GK and VMAT plans,^13^, ^16^ and none of them compared the two treatment modalities with respect to the possibility of delivering FSRT, which is currently rather common for VMAT but not frequent for GK with CTV/PTV margins. Furthermore, they did not report systematic analyses of the performances of the two treatment modalities with respect to the shape and size of the targets. Therefore, it was the aim of this project to present the theoretical performance of fractionated GK treatments with margins making use of the newly introduced patient immobilization system Extend™ and VMAT for large targets that are not subjected to surgery.

One particular challenge of this study on the assessment of the performances of GK and linac‐based treatments for delivering fractionated SRT was the design of the planning methodology in LGP and Eclipse. The methods used in this study when planning in LGP and Eclipse were designed for achieving plans of high quality for both modalities and are the result of methodically exploring the two dose planning systems.

Forward planning in LGP has consequently been used throughout the study. In order to devise the method for planning fractionated treatments using LGP, a prospective study has been performed. Dose plans were created in LGP for the three patients. For each patient, five plans were produced at the normalization isodose levels 30%, 40%, 50%, 60%, and 70% with a requirement of 98% coverage of target. Based on the results of the prospective study, it was observed that, for larger target volumes, dose plans should preferably be made at normalization isodose levels between 35%–60%. At these levels it is possible to simultaneously achieve high coverage, high selectivity, reasonable treatment times, and acceptable GI values. Normalization isodose levels around 60% or higher will lead to difficulties in keeping a low GI and also a risk to increase the equivalent 10 Gy‐volume. Normalization isodose levels around 35% or lower will lead to difficulties in achieving high selectivity at the same time as keeping the treatment time down. The method used in this study is based on the findings of the preliminary study, as well as the current literature.^(6)^ Thus, as suggested by the literature but also observed in the preliminary study, shots should not be placed too close to the edges of the target volume if the aim was a reduced GI.^6^, ^17^ Large, elongated isocenters created by blocking sectors, were also avoided since it has been recognized that they can broaden the dose falloff outside the target.^(17)^ Furthermore, the aim was to always use the largest shots when possible to keep the treatment time on an acceptable level, preferably below 30 min per fraction. For the same reason blocked areas were avoided, if possible.

A similar prospective study for the VMAT plans was also performed for setting up the method for planning in Eclipse. Thus, a series of VMAT plans was made for one of the patients. First, a number of plans were created where one parameter was varied while the others were kept constant. The plan with the smallest 23 Gy‐volume was regarded as the best, and the evaluated parameter was given the associated value in future plans. In the next step, another parameter was varied and evaluated in the same way. The parameters that were varied in this iterative manner were the dose falloff outside PTV (though the normal tissue objectives (NTO)), the maximum dose allowed in the target, the number of arcs, and the optimization time. The other VMAT treatment planning parameters and settings were kept the same, with set values based on literature: collimator rotation, jaw settings, beam energy, MU optimization, priorities for PTV, and priorities for OARs.

The particular set of parameters, given as NTO in Table 4, was selected after evaluating plans made using four sets of values found in the literature,^18^, ^19^ recommended by Varian^(20)^ or previously used at our clinic. The set of values used by Mayo et al.^(19)^ resulted in the best plan and were, therefore, chosen for the rest of the study and reported in Table 4.

In the VMAT optimization, PTV was assigned one lower objective (LO) and two upper objectives (UO). In this study 100% of the PTV volume was set to receive 98% of the prescribed dose with an initial priority of 50 for all plans. The first UO is used to limit the maximum dose in the target by specifying a volume that should receive a maximum dose. Initially, 0% of the PTV volume was set to receive 107% of the prescribed PTV dose with a priority of 50, in accord with the recommendations of the International Commission on Radiation Units and Measurements (ICRU)^(21)^ regarding conformal radiotherapy. The maximum dose that is allowed in the target might influence the dose distribution and, therefore, plans were also made with UO of 102%, as recommended by Varian,^(20)^ 150%, which is commonly used in stereotactic radiotherapy in the clinic, and no dose limit at all. Using an UO of 150% or no dose limit resulted in equally good plans with a smaller 23 Gy‐volume than the plans with other UOs.

It also has to be mentioned that VMAT treatment planning allows much flexibility with respect to the choice of the radiation geometry given by the number and degrees of arcs. In this study, the same radiation geometry was used in all treatment plans. It was chosen because it resulted in a small equivalent 10 Gy‐volume and no consideration was given to the low dose‐bath. Thus, the plans made using one full arc and three half‐arcs resulted in a systematically higher low dose‐bath than the GK plans. It could, therefore, be argued that, by changing the radiation geometry in the VMAT plans selecting different angles and/or blocking more sectors, the low dose‐bath could also be reduced.

The systematic evaluation of the possible planning strategies for the typical SRS targets that preceded the present study allowed us to perform with a fair degree of confidence the comparison between the GK and the VMAT plans. However, it has to be mentioned that treatment plans for GK can potentially be improved by using smaller collimators, but this will result in longer treatment times. For VMAT, treatment planning is even less standardized and the radiation geometry can be chosen, on the basis of individual case, to reduce dose in a certain region or to spread the dose as much as possible. However, based on the planning methods used in this study, the results showed that GK produced better dose distributions for target volumes below 15 cm^3^, while VMAT performed better for larger volumes. For irregularly shaped targets surrounding an OAR, glioma‐like, and for the clinical cases, VMAT spared the OARs to a larger extent than the GK plans.

## V. CONCLUSIONS

In case of fractionated treatments for large or irregular volumes, VMAT planning using one full arc and three half‐arcs results in better dose conformity to the target and lower doses to the OARs. The GK plans performed, however, better in terms of dose distribution to small targets, up to about 15 cm^3^, and resulted in a lower integral dose to the brain. The results of this study could, therefore, lead to clinically significant decisions regarding the choice of the radiotherapy technique for selected brain targets.

## ACKNOWLEDGMENTS

Financial support from the Cancer Research Funds of Radiumhemmet is gratefully acknowledged.
